# Exercise and Carnosine Modulate Microbiota-Derived Metabolites, Myokines, and Cardiometabolic Profiles in Rats: A Randomized Controlled Trial

**DOI:** 10.3390/biomedicines13122853

**Published:** 2025-11-22

**Authors:** Kenan Bozbay, Vedat Çınar, Taner Akbulut, Yavuz Yasul, Mehmet Hanifi Yalçın, Meva Ceren Orgun, Süleyman Aydın, Do-Youn Lee

**Affiliations:** 1 School of Physical Education and Sports, Dicle University, Diyarbakır 21280, Türkiye; kenanbozbay21@gmail.com; 2 Faculty of Sport Sciences, Fırat University, Elazig 23200, Türkiyemcorgun@firat.edu.tr (M.C.O.); 3 Bafra Vocational School, Ondokuz Mayıs University, Samsun 55400, Türkiye; yavuz.yasul@omu.edu.tr; 4 Department of Histology and Embryology, Faculty of Veterinary Medicine, Fırat University, Elazig 23200, Türkiye; 5 Department of Medical Biochemistry, Faculty of Medicine, Fırat University, Elazig 23200, Türkiye; 6 College of General Education, Kookmin University, Seoul 02707, Republic of Korea

**Keywords:** carnosine, exercise, HOMA-IR, IS, lipids, myokines, S-equol, TMAO

## Abstract

**Background/Objectives**: Carnosine and exercise independently improve metabolic health, yet their combined effects on myokines and microbiota-derived metabolites remain underexplored. This study evaluated the synergistic impact of carnosine supplementation and exercise intensity on microbiota-derived metabolites, as well as skeletal muscle and myocardial expression of irisin and myonectin, focusing on lipid and glycemic regulation. **Methods**: A randomized post-test control study was conducted using 49 male Sprague Dawley rats (9 weeks old; 250.39 ± 1.85 g), divided into 7 groups: control (C), sham (S), moderate-intensity continuous training (MICT), high-intensity continuous training (HICT), carnosine (CA), MICT with carnosine (MICT_CA_), and HICT with carnosine (HICT_CA_). Interventions included treadmill-based moderate or high-intensity training and carnosine supplementation (100 mg/kg/day) for 5 weeks. Blood samples were collected post-decapitation; plasma was analyzed for lipid profile, glycemic parameters, and microbiota-derived metabolites using enzymatic and ELISA methods. Irisin and myonectin levels were assessed in plasma and myocardial and skeletal muscle tissues via ELISA and immunohistochemistry. **Results**: The HICT_CA_ group showed the lowest body weight, highest HDL-C, and lowest LDL-C, TC, TG, and atherogenic index. Irisin and myonectin levels in skeletal muscle and myocardium were also highest in HICT_CA_. The trimethylamine N-oxide (TMAO) was lowest and S-equol highest in HICT_CA_, whereas indoxyl sulfate (IS) peaked in HICT and was lowest in the C group. Principal component analysis revealed strong positive associations between HICT_CA_ and cardiometabolic biomarkers. **Conclusions**: High-intensity training combined with carnosine may reduce weight gain, improve lipid and glycemic profiles, and enhance myokines and microbiota-derived metabolites.

## 1. Introduction

Carnosine is a naturally occurring dipeptide synthesized from the amino acids β-alanine and L-histidine. It is present in high concentrations in various tissues, particularly in the gastrointestinal tract, brain, and skeletal muscle [1]. As a non-essential dipeptide, carnosine has garnered considerable scientific interest due to its antioxidant and anti-inflammatory properties. Carnosine plays a pivotal role in cellular defense mechanisms, including the detoxification of reactive oxygen species and the inhibition of protein cross-linking, thereby contributing to the maintenance of intracellular homeostasis [2]. Given its ergogenic potential during high-intensity exercise, carnosine has attracted growing attention as a biomolecule relevant to athletic performance and health. However, the precise biological mechanisms by which carnosine influences skeletal and myocardial muscle function have yet to be fully elucidated [3].

Emerging evidence suggests that, in addition to muscular adaptations, systemic components such as the gastrointestinal tract and gut microbiota may exert a significant influence on athletic performance and overall health. The gut microbiota constitutes a complex and dynamic ecosystem that plays a crucial role in maintaining systemic homeostasis. Evidence indicates that the microbiota can regulate various host physiological functions through the synthesis of short-chain fatty acids and other bioactive metabolites, including trimethylamine N-oxide (TMAO), S-equol, and indoxyl sulfate (IS) [4]. Although factors such as birth mode, age, diet, and antibiotic use are well known to affect microbial composition, the impact of exercise on microbial balance remains poorly understood [5]. Nevertheless, the finding suggests that physical activity may enhance microbial diversity, promote the growth of commensal species, and increase the abundance of beneficial bacterial strains as well as microbiota-derived metabolites [6]. The influence of exercise type, duration, and intensity on microbiota-derived metabolites remains a subject of scientific inquiry, with further research needed to elucidate the implications of these interactions on health outcomes [7,8].

The effects of microbiota-derived metabolites are no longer thought to be limited to the gastrointestinal tract. Emerging evidence indicates that these bioactive compounds may significantly influence both physiological and pathological processes in peripheral organs, including skeletal and myocardial muscle tissues [9]. This paradigm shift in the biological characterization of skeletal muscle is marked by its recognition as a dynamic endocrine organ, challenging the traditional view of muscle as merely a contractile tissue responsible for locomotion. Skeletal muscle can synthesize and release a variety of cytokine-like signaling molecules, collectively referred to as myokines. These molecules possess hormone-like properties and exert systemic effects [10]. Muscle contraction, particularly during physical exertion or regular exercise, acts as a primary stimulus for the secretion of these myokines into the bloodstream. Once released, myokines exert both local and systemic effects, modulating a wide range of biological processes, including energy metabolism, inflammation, and immune regulation [11,12]. Recent research has increasingly focused on irisin and myonectin, two muscle-derived factors that play critical roles in exercise physiology. These factors are of particular interest due to their roles in maintaining muscle homeostasis and improving exercise capacity and metabolic health [13,14]. Evidence suggests that both irisin and myonectin play beneficial roles in metabolic regulation by enhancing glucose uptake, improving insulin sensitivity, and modulating lipid metabolism. However, despite these promising effects, the molecular mechanisms underlying their expression, secretion, and downstream signaling in response to exercise remain poorly understood. This is particularly true regarding their potential interaction with microbiota-derived metabolites, emerging field of research that remains in its early stages but holds substantial promise [15,16].

In this regard, the role of carnosine supplementation in modulating gut microbiota-derived metabolite production under altered exercise intensities, and its association with the expression of myokines such as irisin and myonectin in skeletal and cardiac muscle tissues, has yet to be comprehensively elucidated. Furthermore, the potential impact of these biological interactions on glucose and lipid metabolism, as well as insulin responsiveness, requires further investigation. Therefore, the present hypothesis is that carnosine supplementation, when combined with different exercise intensities, could modulate plasma levels of microbiota-derived metabolites such as TMAO, IS, and S-equol, and tissue-specific levels of irisin and myonectin. It is further postulated that these factors could ultimately influence lipid profiles, glucose concentrations, and insulin responses.

## 2. Materials and Methods

### 2.1. Experimental Design

This study employed a randomized post-test control group design and included 49 male Sprague Dawley rats, each nine weeks old, with a mean body mass of 250.39 ± 1.85. The use of male rats instead of female rats in the study was due to challenges such as controlling hormonal cycles and monitoring the estrous phase. Additionally, there are sex-specific differences in exercise responses [17,18]. The sample size was estimated a priori using G-Power to ensure adequate statistical power and generalizability of the findings. The analysis was performed using a type I error rate (α) of 0.05, statistical power (1 − β) of 0.80, an effect size of 3.18, and a two-tailed alternative hypothesis (H1), based on the parameters reported by Schaalan et al. [19]. The results indicated that a minimum of seven rats per group was required to detect statistically significant differences. Accordingly, the rats were randomly assigned to seven groups: control (C; no intervention), sham (S; saline only), moderate-intensity continuous training (MICT), high-intensity continuous training (HICT), carnosine only (CA), MICT with carnosine (MICTCA), and HICT with carnosine (HICTCA). The animals were housed in standard cages, with three to four rats per cage. The C group received no carnosine or exercise interventions and was fed ad libitum. The S group was administered physiological saline to simulate supplementation, thereby controlling for the independent effects of the administration procedure. The exercise intervention commenced following one-week acclimatization period on the treadmill and continued for 5 weeks, 5 days per week. The design, conduct, and reporting of this animal study were performed in accordance with the ARRIVE (Animal Research: Reporting of In Vivo Experiments) guidelines 2.0, ensuring compliance with internationally recognized standards for methodological transparency and reproducibility in in vivo research.

**Figure 1:**
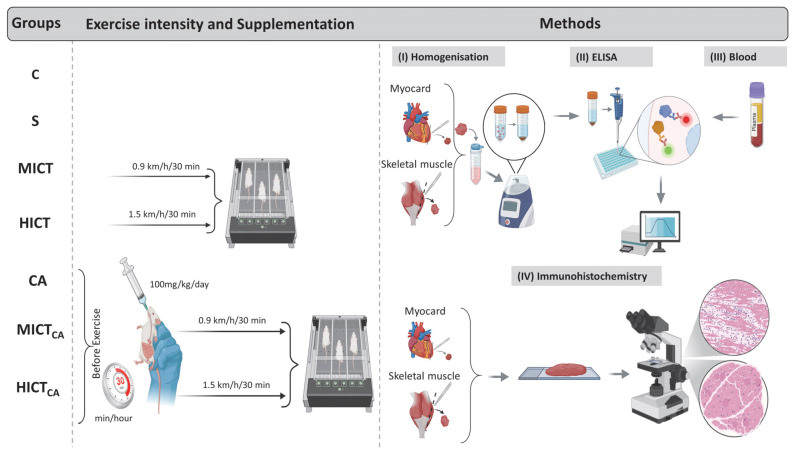
The standardization of exercise intensity, carnosine supplementation (timing and dosage), homogenization (**I**), ELISA procedures (**II**), blood collection (**III**), and immunohistochemical analysis (**IV**) are illustrated. The experimental groups were as follows; C: control group, S: sham group (receiving saline), CA: carnosine supplementation group, MICT: moderate-intensity continuous training group, HICT: high-intensity continuous training group, MICTCA: MICT with combined with carnosine supplementation group, HICTCA: HICT with combined with carnosine supplementation group.

At the end of the experimental period, the rats were euthanized via decapitation, and blood samples were collected into EDTA tubes containing aprotinin. Plasma was separated by centrifugation at 4000 rpm for 5 min at 4 °C. Total cholesterol (TC) and triglyceride (TG) concentrations were determined using enzymatic colorimetric assays. The high-density lipoprotein (HDL) fraction was then separated by precipitation, and HDL cholesterol (HDL-C) was analyzed enzymatically for cholesterol content. Low-density lipoprotein cholesterol (LDL-C) was calculated using the Friedewald formula: LDL-C (mmol/L) = TC − HDL-C − (0.45 × TG). The atherogenic index of plasma (AIP) was calculated as the ratio of TG to HDL-C [20]. Fasting blood samples were collected after a 12 h fast to measure glucose and insulin levels. Glucose concentrations were measured using the hexokinase method on a Beckman AU680 autoanalyzer, while insulin levels were assessed via chemiluminescent microparticle immunoassay (CMIA) using an ARCHITECT i2000SR system [21]. Insulin resistance was calculated using the homeostatic model assessment for insulin resistance (HOMA-IR) formula: HOMA-IR = [fasting insulin (μIU/mL) × fasting glucose (mmol/L)]/405 [22]. Additionally, plasma levels of microbiota-derived metabolites, including TMAO, S-equol, and IS, were measured. The concentrations of the myokines irisin and myonectin were analyzed in homogenized myocardial and skeletal muscle tissues. These analyses were performed using commercially available ELISA kits, according to the manufacturer’s instructions. Immunoreactivity of irisin and myonectin in myocardial and skeletal muscle tissues was further assessed using immunohistochemical methods (Figure 1).

### 2.2. Treadmill-Based Exercise Training

Prior to the initiation of the exercise protocol, rats in the experimental groups (excluding the C, CA, and S groups) underwent a five-day treadmill acclimation period. During this period, the rats ran on a treadmill at a speed of 10 m/min on a 0° incline for 20 min daily [23]. Following the acclimation phase, the rats were assigned to exercise programs of varying intensities. The exercise protocol was designed to minimize stress while ensuring effective training and was implemented according to the principle of progressive overload [24,25,26]. Exercise intensities were organized as follows: for MICT, the treadmill speed was set to 15 m/min (0.9 km/h); for HICT, it was set to 25 m/min (1.5 km/h). Rats in the MICT group ran at 15 m/min for 30 min, whereas those in the HICT group ran at 25 m/min for the same duration. All exercise sessions were performed on a flat-surface (0° incline) KN-73 treadmill (Natsume Seisakusho Co., Ltd., Tokyo, Japan).

### 2.3. Feeding and Supplement Protocol

The rats were provided with standard laboratory chow and given free access to water throughout the study. The standard diet contained 21% protein, 5% fat, 55% carbohydrates, and essential vitamins and minerals. The C group received no intervention. The MICT and HICT groups were subjected exclusively to their respective exercise protocols, while the CA group received carnosine supplementation without exercise. The MICTCA and HICTCA groups received both carnosine supplementation and their respective exercise programs. The literature was reviewed to identify the appropriate dose and formulation of carnosine to be administered to rats [27,28]. Carnosine was administered orally via gavage at a dose of 100 mg/kg/day. The solution was prepared in 1 mL of physiological saline and delivered 30 min prior to each exercise session, five days per week [27,29]. The S group received 1 mL of physiological saline, the vehicle for carnosine, administered in the same manner and timing as the supplementation protocol.

### 2.4. Myocardial and Skeletal Muscle Homogenization

Tissue homogenates were prepared from myocardial and skeletal muscle samples in order to quantify irisin and myonectin levels. Specimens from each rat were excised using sterile scalpels and weighed to exactly 50 mg using an analytical balance. Each sample was transferred to a pre-labeled Eppendorf tube containing 4–5 homogenization beads (3.2 mm diameter; total weight ~ 500 mg) and 450 µL of phosphate-buffered saline (PBS). The tubes were tightly sealed and homogenized using a Bullet Blender (Next Advance Inc., Troy, NY, USA) at speed level 8 for 5 min. The homogenate was transferred to a new Eppendorf tube and centrifuged at 4 °C for 5 min at 4000 rpm using a refrigerated centrifuge (Beckman Coulter, Allegra^®^ X-30; Brea, CA, USA). The supernatant was carefully collected and stored at −80 °C to preserve sample integrity for subsequent analysis of irisin and myonectin concentrations. Irisin and myonectin levels were converted from pg/mL to ng/mg to ensure comparability of the analyses. 

### 2.5. ELISA

Plasma concentrations of microbiota-derived metabolites, TMAO, S-equol, and indoxyl sulfate (IS), were quantified using enzyme-linked immunosorbent assay. The detection ranges, sensitivities, and catalog numbers were as follows: TMAO (15.63–1000 ng/mL; sensitivity: 4.93 ng/mL, Cat No; RE10066; Reed Biotech Ltd., Wuhan, China), S-equol (0.25–70 ng/mL; 0.251 ng/mL, Cat No; 201-11-6639; Sun-Red Bio Company, Shanghai, China), and IS (31.25–2000 ng/mL; 18.75 ng/mL, Cat No; RE10180; Reed Biotech Ltd., Wuhan, China). A similar approach was used to determine tissue levels of irisin and myonectin in myocardial and skeletal muscle homogenates. For both biomarkers, the detection range was 78.13–5000 ng/mL, with a sensitivity of 46.88 ng/mL. The catalog numbers were (Cat No; RE2836R; Reed Biotech Ltd., Wuhan, China) for irisin and (Cat No; RE3549R; Reed Biotech Ltd., Wuhan, China) for myonectin. All measurements were performed in strict accordance with the protocols provided by the respective kit manufacturers. Automated plate washing was performed using a Bio-Tek ELX50 microplate washer (BioTek Instruments, Winooski, VT, USA). Absorbance values at 450 nm were measured using a ChroMate Microplate Reader P4300 (Awareness Technology Instruments, Palm City, FL, USA).

### 2.6. Immunohistochemistry

Tissue samples were stained according to the immunohistochemical protocol described by Hsu et al. [30]. Specimens were fixed in 10% neutral buffered formalin at room temperature for 24 h, dehydrated through a graded ethanol series, cleared in xylene, and embedded in paraffin. Paraffin-embedded blocks were sectioned at 4–6 µm using a Leica RM2125 microtome (Leica Biosystems, Buffalo Grove, IL, USA). Sections were mounted on poly-L-lysine-coated slides and dried at 80 °C for 15 min. They were deparaffinized in xylene (three times for 5 min), rinsed in water, and rehydrated through a descending ethanol series. Antigen retrieval was performed by heating sections in 0.01 M sodium citrate buffer (pH 6.0) in a microwave at 94–100 °C for 7.5 min, followed by 20 min of cooling at room temperature. Slides were washed in PBS (P4417, Sigma-Aldrich, St. Louis, MO, USA) three times for 5 min. To block endogenous peroxidase activity and reduce nonspecific staining, hydrogen peroxide block and Ultra V Block solutions (Thermo Fisher Scientific/Lab Vision, Fremont, CA, USA) were sequentially applied. Primary antibodies targeting mouse irisin and myonectin (CTRP5, a member of the myonectin/CTRP family) were incubated at room temperature for 60 min. PBS was used as the negative control in place of the primary antibody. Slides were then incubated with a biotin-conjugated anti-mouse/rabbit IgG secondary antibody (TP–125-BN; Lab Vision Corp.) and washed three times with PBS. Staining was performed using 3-amino-9-ethylcarbazole (AEC) chromogen (Lab Vision Corp., Fremont, CA, USA) at 38 °C for 10 min, followed by counterstaining with Mayer’s hematoxylin for 1–2 min. Stained sections were examined and imaged using a Leica DM500 microscope (Wetzlar, Germany) [31].

### 2.7. Statistical Analysis

The statistical analyses were performed using IBM SPSS Statistics 21 (Armonk, NY, USA). The Shapiro–Wilk test was used to assess the normality of data distribution. Changes in body mass were analyzed using two-way analysis of variance, with Geisser–Greenhouse correction applied when the assumption of sphericity was violated. Post hoc comparisons were performed using Sidak’s multiple comparisons test. Also, between-group comparisons were performed using one-way ANOVA to assess plasma lipid concentrations, glycemic markers, tissue levels of irisin and myonectin, and microbiota-derived metabolite concentrations, with Scheffé’s test applied for post hoc analyses. Data are presented as mean ± standard deviation (SD). Multivariate data structure was explored using Principal Component Analysis (PCA), performed with the trial version of XLSTAT software (version 2019; Addinsoft, New York, NY, USA) on a correlation matrix, retaining components with eigenvalues ≥ 1. This analysis enabled the explanation of biomarker-based variance and the identification of intergroup differentiation patterns [32,33,34]. Sequential multiple mediation analysis (PROCESS Model 6, SPSS 21 macro) was conducted to investigate the indirect effects of group status on lipid, glycemic, and microbiota-derived parameters, with irisin and myonectin included as sequential mediators [35]. Effect sizes were calculated using eta-squared (η^2^), and interpreted according to Cohen’s criteria as small (η^2^ = 0.0099), medium (η^2^ = 0.0588), and large (η^2^ = 0.1379) [36].

### 2.8. Ethical Approval

The experimental procedures were conducted at the Laboratory Animal Research Center of Fırat University in accordance with the National Institutes of Health (NIH) guidelines for the care and use of laboratory animals. Ethical approval was granted by the Ethics Committee for Animal Experiments at Fırat University on 16 December 2022 (Approval No: 12263/18-05).

## 3. Results

**Figure 2:**
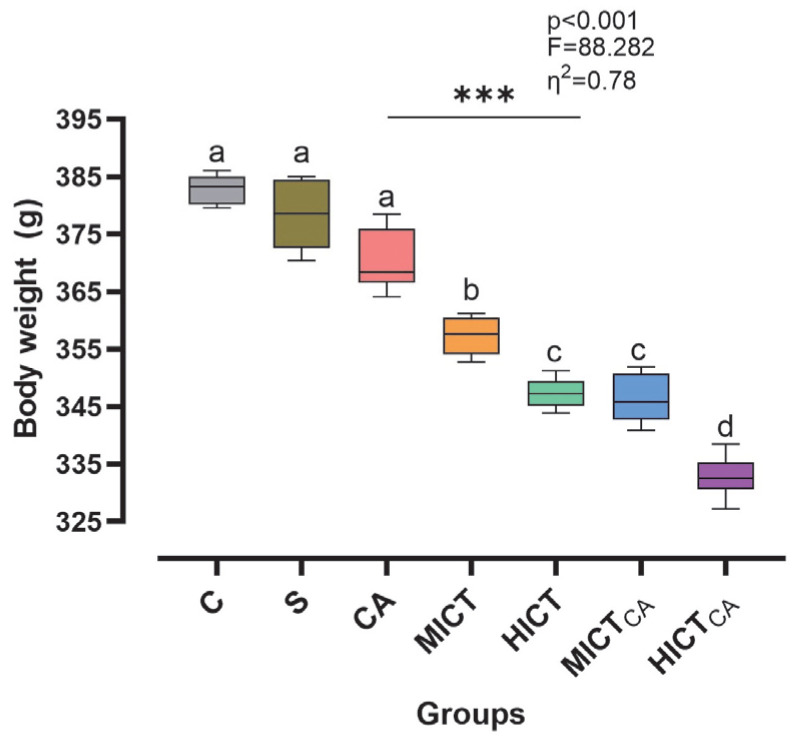
The changes in body mass are illustrated. Superscript letters above the bars (a–d) indicate statistically significant differences among groups; bars sharing the same letter are not significantly different. The experimental groups were as follows; C: control group, S: sham group (receiving saline), CA: carnosine supplementation group, MICT: moderate-intensity continuous training group, HICT: high-intensity continuous training group, MICTCA: MICT with combined with carnosine supplementation group, HICTCA: HICT with combined with carnosine supplementation group. Data are presented as mean ± standard deviation (SD), η^2^: eta squared, *** *p* < 0.001.

According to Figure 2, the body mass levels of the groups at the baseline stage of the experimental intervention demonstrated a homogeneous structure, with no statistically significant differences observed (*p* > 0.05). However, post-test measurements revealed statistically significant and substantial difference in body mass among the groups (*p* < 0.001, F = 88.282, η^2^ = 0.78). Furthermore, the effect of time on this difference was found to be both significant and pronounced (*p* < 0.001, η^2^ = 0.98). In terms of the impact of the experimental groups, it was determined that the administration of different exercise intensities in combination with carnosine supplementation exerted a statistically significant and substantial effect on body mass (*p* < 0.001, η^2^ = 0.88). When the post-test groups were compared, the HICTCA group demonstrated the lowest increase in body mass, followed by the HICT and MICTCA groups, respectively, when compared to the C and S groups. Collectively, these findings indicate that the time × group interaction was statistically significant (*p* < 0.001, η^2^ = 0.76), demonstrating that changes in body mass varied significantly across groups over time.

**Figure 3:**
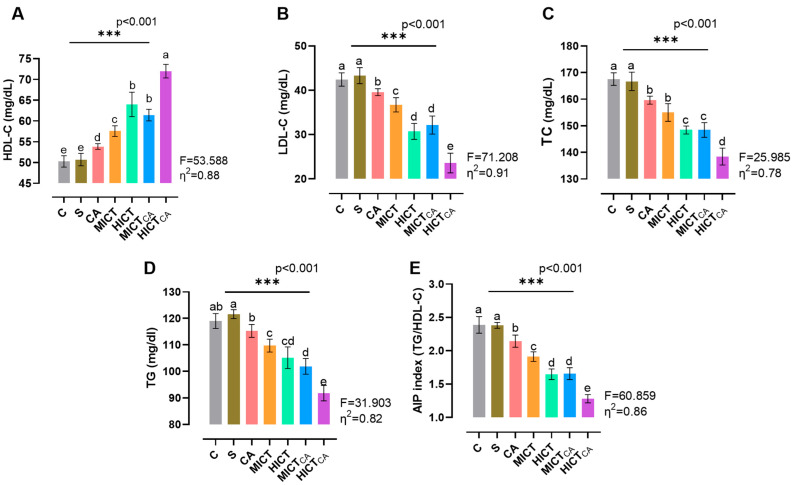
The lipid levels of plasma (**A**–**D**) and the AIP index (**E**) are illustrated. Superscript letters above the bars (a–e) indicate statistically significant differences among groups; bars sharing the same letter are not significantly different. HDL-C: high-density lipoprotein cholesterol, LDL-C: low-density lipoprotein cholesterol, TC: total cholesterol, TG: triglyceride, AIP: atherogenic index of plasma. The experimental groups were as follows; C: control group, S: sham group (receiving saline), CA: carnosine supplementation group, MICT: moderate-intensity continuous training group, HICT: high-intensity continuous training group, MICTCA: MICT with combined with carnosine supplementation group, HICTCA: HICT with combined with carnosine supplementation group. Data are presented as mean ± standard deviation (SD). Between-group comparisons were conducted using one-way ANOVA, followed by Scheffé’s post hoc test, η^2^: eta squared, *** *p* < 0.001.

According to Figure 3A, there was statistically significant difference in HDL-C levels among the groups (F = 53.588, *p* < 0.001). The highest HDL-C concentration was observed in the HICTCA group, particularly when compared to the C and S groups, as well as the other intervention groups (*p* < 0.001). Furthermore, MICTCA and HICT groups exhibited significantly higher HDL-C levels compared to the C, S and CA groups. The MICTCA and HICT groups were found to be in the same statistical group (*p* = 0.113). According to Figure 3B, while LDL-C levels exhibited significant differences between the groups (F = 71.208, *p* < 0.001), the lowest LDL-C levels were observed in the HICTCA group compared to the other groups, particularly in groups C and S (*p* < 0.001). The MICTCA group and the HICT group, which had significantly lower LDL-C levels compared to the C group, S group and CA group, were in the same statistical group and had similar LDL-C levels (*p* = 0.885). According to Figure 3C, while the TC level differed significantly between the groups (F = 25.985, *p* < 0.001), the lowest TC level was found in the HICTCA group compared to the other groups, especially the C and S groups (*p* < 0.001). The MICTCA and HICT groups, which exhibited significantly lower TC levels in comparison to the C, S, and CA groups, were found to be in the same statistical group and demonstrated comparable TC levels (*p* = 0.998). According to Figure 3D, while TG levels exhibited significant differences between the groups (F = 31.903, *p* < 0.001), the lowest TG levels were observed in the HICTCA group compared to the other groups, particularly the C and S groups (*p* < 0.001). The MICTCA and HICT groups exhibited significantly lower TG levels compared to the C, S, and CA groups. However, the MICTCA and HICT groups were found to be in the same statistical group and had similar TG levels (*p* = 0.864). Furthermore, the TG level was relatively higher in the MICT group compared to the HICT group, but statistically in the same group (*p* = 0.581). Furthermore, the TG level was relatively higher in the MICT group compared to the HICT group, but statistically in the same group (*p* = 0.581). According to Figure 3E, while the AIP index differed significantly between the groups (F = 60.859, *p* < 0.001), the lowest AIP index was found in the HICTCA group compared to the other groups, especially the C and S groups (*p* < 0.001). The MICTCA group and the HICT group, which exhibited a significantly lower AIP index compared to the C and S groups, were observed to be in the same statistical group and had a similarly low AIP index (*p* = 0.993). Furthermore, although the TG level was relatively higher in the MICT group compared to the HICT group, the two groups were in the same statistical group (*p* = 0.581).

**Figure 4:**
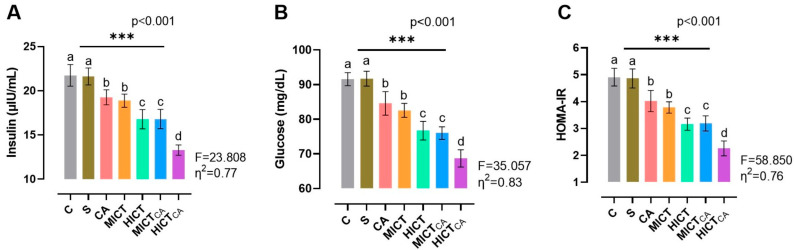
The insulin (**A**) and glucose levels (**B**), along with the HOMA-IR index (**C**), are illustrated. Superscript letters above the bars (a–d) indicate statistically significant differences among groups; bars sharing the same letter are not significantly different. HOMA-IR: homeostatic model index. The experimental groups were as follows; C: control group, S: sham group (receiving saline), CA: carnosine supplementation group, MICT: moderate-intensity continuous training group, HICT: high-intensity continuous training group, MICTCA: MICT with combined with carnosine supplementation group, HICTCA: HICT with combined with carnosine supplementation group. Data are presented as mean ± standard deviation (SD). Between-group comparisons were conducted using one-way ANOVA, followed by Scheffé’s post hoc test, η^2^: eta squared, *** *p* < 0.001.

According to Figure 4A, there was significant difference in insulin levels between the groups (F = 23.808, *p* < 0.001). It was observed that the HICTCA group exhibited the lowest insulin levels when compared with the other groups, particularly the C and S groups (*p* < 0.001). The MICTCA and HICT groups, which exhibited significantly lower insulin levels compared to the C, S, and CA groups, were in the same statistical group and had similar insulin levels (*p* = 0.978). According to Figure 4B, there was significant difference in glucose levels between the groups (F = 35.057, *p* < 0.001). The lowest glucose level was observed in the HICTCA group compared to the other groups, particularly in the C and S groups (*p* < 0.001). Furthermore, the MICTCA and HICT groups exhibited significantly lower glucose levels in comparison to the C, S, and CA groups. The MICTCA and HICT groups were observed to be in the same statistical group and had similar insulin levels (*p* = 0.968). According to Figure 4C, there was significant difference between the groups in terms of the HOMA-IR index (F = 58.850, *p* < 0.001). The lowest HOMA-IR index level was found in the HICTCA group in comparison to the other groups, especially in the C and S groups (*p* < 0.001). Additionally, the MICTCA and HICT groups, which exhibited significantly lower HOMA-IR index levels in comparison to the C, S, and CA groups, were observed to be in the same statistical group and exhibited similar insulin levels (*p* > 0.999).

**Figure 5:**
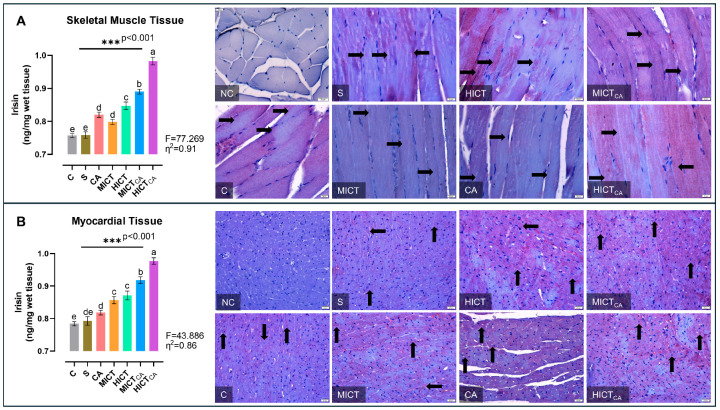
The irisin levels in skeletal muscle tissue (**A**) and myocardial tissue (**B**), presented as column bar graphs, along with cellular immunoreactivity shown in histological images marked with black arrows, are illustrated. Tissue sections were visualized via immunohistochemical staining employing AEC chromogen and counterstained with Mayer’s hematoxylin. Superscript letters above the bars (a–e) indicate statistically significant differences among groups; bars sharing the same letter are not significantly different. The experimental groups were as follows; C: control group, S: sham group (receiving saline), CA: carnosine supplementation group, MICT: moderate-intensity continuous training group, HICT: high-intensity continuous training group, MICTCA: MICT with combined with carnosine supplementation group, HICTCA: HICT with combined with carnosine supplementation group. Data are presented as mean ± standard deviation (SD). Between-group comparisons were conducted using one-way ANOVA, followed by Scheffé’s post hoc test, η^2^: eta squared, *** *p* < 0.001.

According to Figure 5A, there was significant difference in irisin levels in skeletal muscle homogenates between the groups (F = 77.269, *p* < 0.001). The highest irisin levels were observed in the HICTCA group, particularly compared to the C and S groups. The second highest irisin level was found in the MICTCA group and the third highest irisin level was recorded in the HICT group (*p* < 0.001). The MICT and CA groups, which had significantly higher levels of irisin than the C and S groups, were in the same statistical group and had similar levels of irisin (*p* = 0.643). Furthermore, irisin positivity was concentrated in darkly stained A bands in longitudinal sections, which was revealed by immunohistochemical examination of skeletal muscle tissue. When the groups were compared, the lowest level of irisin expression was observed in the C and S groups, while the highest level was noted in the HICTCA group. Additionally, irisin positivity increased remarkably in the CA, MICT, HICT and MICTCA groups compared to the C and S groups. According to Figure 5B, there were significant differences in irisin levels in myocardial homogenates between the groups (F = 43.886, *p* < 0.001). The highest irisin levels were observed in the HICTCA group, followed by the MICTCA group and then the HICT group, particularly compared to the C and S groups (*p* < 0.001). The MICT and HICT groups, which had significantly higher irisin levels compared to the C and S groups, were in the same statistical group and had similar irisin levels (*p* = 0.960). Furthermore, irisin positivity was observed in myocardial tissue by immunohistochemical analysis. When the groups were compared, the lowest irisin positivity was detected in the C and S groups, followed relatively by the CA group, whereas the highest expression was detected in the HICTCA group. Additionally, irisin positivity increased significantly in the MICT, HICT and MICTCA groups compared to the C, S and CA groups.

**Figure 6:**
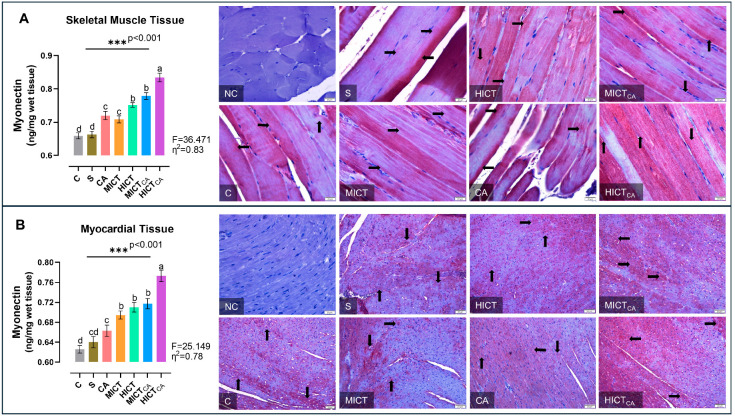
The myonectin levels in skeletal muscle tissue (**A**) and myocardial tissue (**B**), presented as column bar graphs, along with cellular immunoreactivity shown in histological images marked with black arrows, are illustrated. Tissue sections were visualized via immunohistochemical staining employing AEC chromogen and counterstained with Mayer’s hematoxylin. Superscript letters above the bars (a–d) indicate statistically significant differences among groups; bars sharing the same letter are not significantly different. The experimental groups were as follows; C: control group, S: sham group (receiving saline), CA: carnosine supplementation group, MICT: moderate-intensity continuous training group, HICT: high-intensity continuous training group, MICTCA: MICT with combined with carnosine supplementation group, HICTCA: HICT with combined with carnosine supplementation group. Data are presented as mean ± standard deviation (SD). Between-group comparisons were conducted using one-way ANOVA, followed by Scheffé’s post hoc test, η^2^: eta squared, *** *p* < 0.001.

According to Figure 6A, myonectin levels in skeletal muscle homogenates exhibited significant differences between groups (F = 36.471, *p* < 0.001). The HICTCA group indicated the highest myonectin levels, particularly compared to the C and S groups. The second highest myonectin levels were observed in the MICTCA and HICT groups, while the third highest myonectin levels were detected in the CA and MICT groups (*p* < 0.001). The MICT and CA groups, which had significantly higher myonectin levels compared to the C group and S group, were in the same statistical group and had similar myonectin levels (*p* = 0.986). Furthermore, myonectin positivity was concentrated in darkly stained A bands in longitudinal skeletal muscle tissue sections. The C and S groups exhibited the lowest myonectin positivity, while the HICTCA group exhibited the highest. Additionally, the CA and MICT groups, as well as the HICT and MICTCA groups, exhibited higher levels of myonectin positivity than the C and S groups. According to Figure 6B, myonectin levels in myocardial homogenates exhibited significant differences between groups (F = 25.149, *p* < 0.001). The HICTCA group indicated the highest myonectin levels, particularly compared to the C and S groups. The second highest myonectin levels were observed in the MICTCA, HICT ve MICT groups. The MICTCA, HICT, and MICT groups exhibited significantly elevated myonectin levels compared to the C, S, and CA groups, and were observed to be in the same statistical group (*p* < 0.001). Additionally, myonectin positivity was observed in myocardial tissue cells through immunohistochemical examination. The lowest myonectin positivity was detected in the C and S groups, while the highest expression was observed in the HICTCA group compared to the other groups.

**Figure 7:**
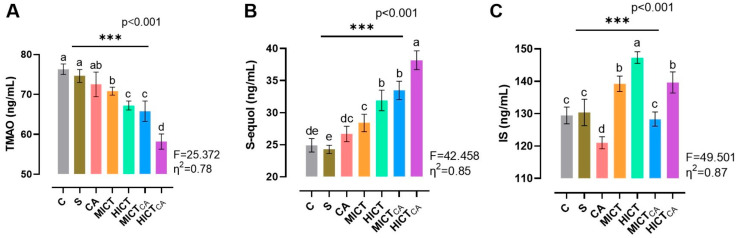
The microbiota-derived metabolites TMAO (**A**), S-equol (**B**) and IS (**C**) in plasma are illustrated. Superscript letters above the bars (a–e) indicate statistically significant differences among groups; bars sharing the same letter are not significantly different. TMAO: trimethylamine N-oxide, IS: indoxyl sulfate. The experimental groups were as follows; C: control group, S: sham group (receiving saline), CA: carnosine supplementation group, MICT: moderate-intensity continuous training group, HICT: high-intensity continuous training group, MICTCA: MICT with combined with carnosine supplementation group, HICTCA: HICT with combined with carnosine supplementation group. Data are presented as mean ± standard deviation (SD). Between-group comparisons were conducted using one-way ANOVA, followed by Scheffé’s post hoc test, η^2^: eta squared, *** *p* < 0.001.

According to Figure 7A, while there was significant difference in TMAO levels between the groups (F = 25.372, *p* < 0.001), the lowest TMAO levels were observed to be in the HICTCA group compared to other groups, particularly the C and S groups (*p* < 0.001). Furthermore, the MICTCA and HICT groups, which exhibited significantly lower TMAO levels compared to the C and S groups, were observed to be in the same statistical group (*p* = 0.993). These groups also demonstrated relatively similar TMAO levels. According to Figure 7B, while there was significant difference in S-equol levels between the groups (F = 42.458, *p* < 0.001), the highest S-equol levels were observed in the HICTCA group compared to other groups, particularly the C and S groups (*p* < 0.001). Additionally, the MICTCA and HICT groups, which exhibited the highest levels of S-equol compared to the C and S groups, were found to be in the same statistical groups (*p* = 0.731). According to Figure 7C, while there was significant difference in IS levels between the groups (F = 49.501, *p* < 0.001), the highest IS levels were observed in the HICT groups compared to other groups, particularly the C and S groups (*p* < 0.001). Moreover, the HICTCA and MICT groups exhibited significantly higher IS levels than the C and S groups.

**Figure 8:**
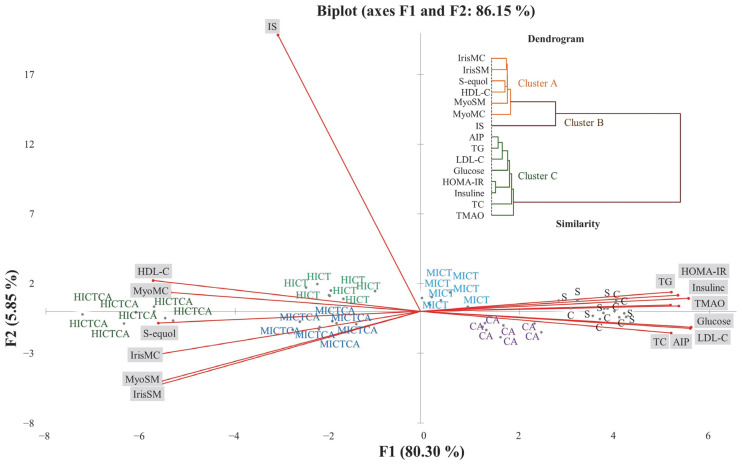
The distribution of intergroup variance and separation patterns in plasma, myocardial, and skeletal muscle tissues were illustrated in the PCA biplot and analyzed to explore the data structure and intergroup relationships. The experimental groups were as follows; C: control group, S: sham group (receiving saline), CA: carnosine supplementation group, MICT: moderate-intensity continuous training group, HICT: high-intensity continuous training group, MICTCA: MICT with combined with carnosine supplementation group, HICTCA: HICT with combined with carnosine supplementation group. IrisMC and MyoMC refer to irisin and myonectin levels in myocardiaal tissue, while IrisSM and MyoSM refer to irisin and myonectin levels in skeletal muscle homogenate. HDL-C: high-density lipoprotein cholesterol, LDL-C: low-density lipoprotein cholesterol, TC: total cholesterol, TG: triglyceride, AIP: atherogenic index of plasma, HOMA-IR: homeostatic model index, TMAO: trimethylamine N-oxide, IS: indoxyl sulfate.

As shown in Figure 8, principal component analysis (PCA) revealed distinct patterns of variance that explain the biomarker-based distribution and characterize intergroup separation profiles. These results delineate the multivariate distribution of biomarkers and the intergroup relationships present within the dataset. The first two principal components accounted for 86.15% of the total variance, while the first three components with eigenvalues ≥ 1 explained 88.82%. The main contributors to the first principal component (F1) were AIP (7.59%), LDL-C (7.54%), HOMA-IR (7.45%), HDL-C (7.42%), S-equol (7.13%), and IrisSM (7.01%), followed by MyoSM (6.96%), IrisMC (6.95%), glucose (6.92%), TG (6.87%), insulin (6.52%), TC (6.51%), myonectinMC (6.48%), TMAO (6.48%), myonectinSM (6.48%), and IS (2.11%). The second principal component (F2) was mainly influenced by IrisSM (5.81%), myonectinSM (5.30%), IrisMC (1.96%), and HDL-C, along with LDL-C (0.31%), TC (0.50%), TG (0.28%), insulin (0.26%), glucose (0.40%), HOMA-IR (0.03%), myonectinMC (0.18%), TMAO (0.38%), S-equol (0.04%), and IS (0.15%). The HICT and MICT exercise groups, located near the origin (0,0), together with the MICTCA and CA groups, displayed relatively higher levels of metabolic adaptation indicators. The C and S groups were positioned in proximity to TMAO and vectors representing cardiometabolic risk-associated variables, including TC, TG, LDL-C, AIP, glucose, insulin, and HOMA-IR. Conversely, the HICTCA and MICTCA groups were more closely related to HDL-C, irisin, myonectin (both skeletal muscle and myocardial), and S-equol. IS was positioned in the upper quadrant of the biplot, distinctly separated from other variables and groups, suggesting a relative independence from systemic metabolic load. It is important to note that these spatial associations are correlational in nature and do not imply causality.

**Figure 9:**
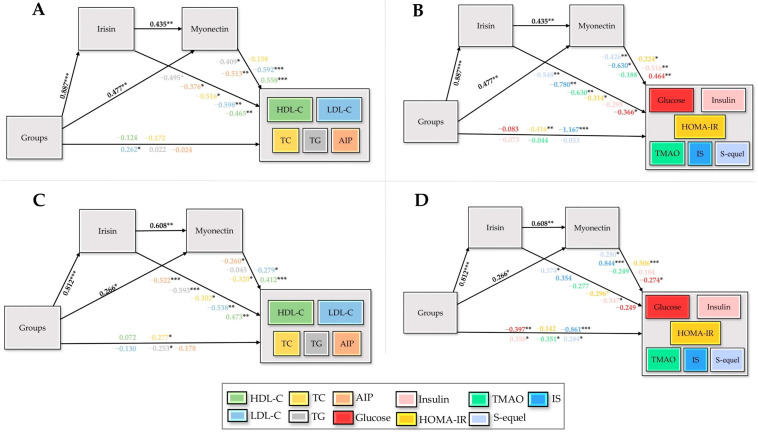
Structural equation models depicting the effects of groups (X) on irisin (M1) and myonectin (M2) derived from skeletal muscle (**A**,**B**) and myocardial tissue (**C**,**D**), and their downstream influences on lipid markers, glycemic indicators, and microbiota-related metabolites, with standardized path coefficients and significance levels indicated, are illustrated. HDL-C: high-density lipoprotein cholesterol, LDL-C: low-density lipoprotein cholesterol, TC: total cholesterol, TG: triglycerides, AIP: atherogenic index of plasma, HOMA-IR: homeostatic model assessment of insulin resistance, TMAO: trimethylamine-N-oxide, IS: indoxyl sulfate, X: independent variable (groups), Y: dependent variables, M1/M2: sequential mediators, *: *p* < 0.05, **: *p* < 0.01, ***: *p* < 0.001.

According to Figure 9A,B, skeletal muscle tissue exhibited a pronounced group influence on irisin (β = 0.887, *p* < 0.001) and a significant effect on myonectin (β = 0.477, *p* < 0.01). Irisin and myonectin in this tissue exerted significant impacts on HDL-C, LDL-C, TG, TC, AIP, glucose, insulin, HOMA-IR, TMAO, IS, and S-equol; however, the effects of myonectin on TC and TMAO were not significant. The direct contributions of groups to LDL-C, HOMA-IR, and IS were also minimal, indicating that the overall effects were predominantly mediated through myokine-dependent pathways. According to Figure 9C,D, myocardial tissue exhibited a substantial group influence on irisin (β = 0.812, *p* < 0.001) and a significant effect on myonectin (β = 0.266, *p* < 0.05). Irisin exerted significant effects on S-equol, HOMA-IR, and insulin, whereas myonectin significantly influenced S-equol, IS, HOMA-IR, and glucose. The direct contributions of groups to TC, TG, glucose, insulin, TMAO, IS, and S-equol were also significant. The myocardial tissue, the overall effects were predominantly mediated through myonectin-dependent pathways, with group influences primarily channeled via this mediator (Table S1).

## 4. Discussion

High or moderate-intensity exercise with carnosine supplementation produced coordinated improvements in lipid and glycemic metabolism, enhanced myokine release, and modulated microbiota-derived metabolites. This integrated approach may offer a practical strategy for cardiometabolic optimization. Exercise is a key modulator of lipid metabolism, driving skeletal muscle adaptation, adiposity reduction, and favorable lipoprotein remodeling [37,38,39]. High-intensity protocols induce rapid metabolic adaptations, whereas moderate-intensity exercise yields gradual but sustained lipid benefits [40]. The HICTCA group exhibited the most favorable lipid profile, characterized by increased HDL-C and reduced LDL-C, TC, and TG, accompanied by minimal body-mass gain. AIP reduction was likewise evident in the HICT, MICTCA, and HICTCA groups. PCA revealed vector alignment of HDL-C and S-equol with the skeletal and myocardial myokines irisin and myonectin, demonstrating a coordinated regulatory pattern connecting lipid metabolism, myokine signaling, and microbiota-derived metabolites. Consistent with this integrative regulation, mediation analysis confirmed that group-related alterations in HDL-C, LDL-C, TC, TG, and AIP were primarily transmitted through irisin and myonectin, indicating a myokine-dependent mediation of lipid outcomes. These findings align with previous evidence reporting comparable lipid improvements following exercise [41] or carnosine supplementation [42]. Carnosine appears to modulate glucose homeostasis via both central and peripheral mechanisms. In normoglycemic rats, activation of central histamine H_3_ receptors has been shown to attenuate sympathetic outflow and suppress hepatic gluconeogenesis, thereby reducing endogenous glucose production [43,44]. Peripherally, carnosine may promote glucose uptake in skeletal muscle and adipose tissue, and support insulin signaling through its antioxidant and anti-glycation properties [45,46]. These effects together suggest that exercise and carnosine supplementation may potentially contribute to improving glycemic balance, although the precise molecular pathways have not yet been fully elucidated [47,48,49,50,51,52].

High-intensity exercise combined with carnosine supplementation produced the strongest improvements in glycemic regulation, particularly in the HICTCA group, which exhibited lower HOMA-IR, glucose, and insulin concentrations. These findings indicate a clear enhancement of insulin sensitivity and glucose homeostasis. Hoseini et al. [53] demonstrated improved insulin signaling following structured exercise, while Gallo-Villegas et al. [54] reported comparable benefits across varying intensities. Previous studies confirmed that endurance and resistance training improve insulin sensitivity [55,56], and carnosine supplementation has been shown to lower glucose levels [57,58,59]. Reduction in adiposity is associated with improved insulin sensitivity and modulation of myokine expression [60,61], while remodeling of the gut microbiota may enhance energy homeostasis [62]. Carnosine’s anti-glycation, antioxidant, and anti-inflammatory properties further support glucose regulation independent of body-composition changes [63,64], possibly via suppression of advanced glycation end-products and preservation of insulin signaling [65]. Mediation analysis revealed that group-related changes in glucose, insulin, and HOMA-IR were primarily mediated by irisin and myonectin in both skeletal and myocardial tissues. The skeletal muscle, irisin and myonectin exhibited a clear dominance, reflecting their parallel and coordinated roles in glycemic regulation. In contrast, myonectin demonstrated a more prominent mediating effect in myocardial tissue, particularly influencing glucose and insulin responses. Collectively, these findings suggest that improvements in glycemic control were largely driven by myokine-mediated mechanisms, with tissue-specific predominance of both irisin and myonectin in skeletal muscle and a dominant role for myonectin in the myocardium.

Recent evidence highlights the modulatory role of exercise on gut microbiota and its implications for metabolic health, with studies showing that microbial composition influences gastrointestinal barrier function, exercise performance, and skeletal muscle adaptation. Among microbiota-derived metabolites, TMAO has been identified as a key marker associated with cardiometabolic risk and potentially relevant to exercise physiology [66,67]. PCA revealed that TMAO aligned closely with cardiometabolic indicators such as TC, TG, LDL-C, AIP, glucose, insulin, and HOMA-IR, particularly in the sedentary C and S groups, reflecting a metabolic profile consistent with low activity and elevated cardiometabolic risk. These results align with Taesuwan et al. [68], who reported intramuscular TMAO accumulation reflecting reduced metabolic utilization under inactivity. In contrast, the MICT, HICT, MICTCA, and HICTCA groups exhibited markedly lower TMAO concentrations together with higher S-equol levels, consistent with evidence that regular exercise promotes anti-inflammatory and antioxidative microbial metabolites [69]. PCA confirmed that, in the C and S groups, TMAO clustered with cardiometabolic markers—including TC, TG, LDL-C, AIP, glucose, and HOMA-IR—demonstrating its close relationship with systemic metabolic stress. Previous studies have described transient TMAO reductions after acute exercise [70,71], whereas the present data suggest a more sustained decrease with long-term training. Mediation analysis demonstrated that TMAO was significantly influenced by irisin within skeletal muscle, reflecting a tissue-specific regulatory pattern. In myocardial tissue, TMAO was not associated with myokine-mediated effects but was instead directly modulated via the group effect, indicating a distinct regulatory mechanism. The CA group, receiving supplementation alone, showed minimal change and remained aligned with risk-associated parameters, reinforcing evidence that nutritional supplementation without exercise exerts limited influence on TMAO metabolism [72].

S-equol concentrations reached their highest levels in the HICTCA group, accompanied by elevated HDL-C, irisin, and myonectin concentrations. This profile suggests that high-intensity exercise combined with carnosine supplementation enhances the formation of S-equol, a microbiota-derived metabolite with recognized anti-inflammatory and vasoprotective properties [73]. Produced through microbial biotransformation of soy isoflavones, S-equol has been linked to modulation of endothelial function and attenuation of inflammatory signaling cascades [74]. PCA further revealed that S-equol closely with HDL-C and the skeletal muscle–derived myokines irisin and myonectin, suggesting a coordinated regulatory interaction between microbial metabolites and muscle-derived signaling. Higher S-equol concentrations were inversely associated with glucose, insulin, and HOMA-IR, suggesting improved glycemic regulation consistent with previous reports on metabolic homeostasis [75]. Mediation analysis confirmed that S-equol was significantly influenced by both irisin and myonectin in skeletal muscle, whereas in myocardial tissue, the effect was weaker and predominantly associated with myonectin activity, reflecting a tissue-specific divergence in myokine-mediated regulation. S-equol concentrations were also elevated in the CA group, indicating that supplementation alone may contribute to a moderate rise in this metabolite, while the combination with exercise further amplified this effect. The strong association between S-equol and HDL-C reinforces its proposed role in lipid modulation and endothelial integrity [76].

IS concentrations exhibited marked intergroup variation, with the highest levels observed under sustained high-intensity exercise, particularly in the HICT and HICTCA groups. PCA demonstrated that IS clustered distinctly from other metabolic indicators, indicating a low susceptibility to modulation through exercise or supplementation. This observation aligns with its established characterization as a gut microbiota–derived uremic toxin originating from microbial tryptophan metabolism and defined by strong plasma protein binding, limited renal clearance, and prolonged systemic retention [77,78]. Structural equation modeling further revealed that in skeletal muscle, IS was indirectly regulated through irisin and myonectin, while direct group effects were minimal, suggesting a predominantly myokine-mediated pathway. Myocardial tissue, IS was significantly affected by myonectin, with additional direct group contributions, indicating tissue-specific regulation of IS under metabolic stress. These observations suggest that IS accumulation is a downstream reflection of metabolic strain rather than a directly modifiable variable, aligning with evidence of its association with oxidative stress, mitochondrial dysfunction, and impaired antioxidant defense [79,80,81].

### Limitations

The experimental design incorporated a single exercise protocol based on continuous treadmill running, which limits extrapolation to other training modalities such as interval or resistance training, each of which may elicit distinct physiological adaptations. The five-week intervention reflects short-term responses and does not permit evaluation of long-term effects. Additionally, the absence of baseline lipid and glucose data constrains interpretation of temporal metabolic changes. Carnosine was administered at a single fixed dose without evaluation of potential dose–response relationships; thus, pharmacokinetic analyses are warranted to determine systemic bioavailability and optimize dosing strategies. Differentiating additive from synergistic interactions between exercise and carnosine supplementation was beyond the scope of the current design. Despite these limitations, the study offers a robust experimental foundation for future investigations into the integrated metabolic effects of carnosine supplementation combined with structured exercise.

## 5. Conclusions

This study demonstrated that the interaction between exercise intensity and carnosine supplementation produced differential effects on cardiometabolic regulation across the intervention groups. The HICTCA group exhibited the most favorable overall profile, with significant reductions in LDL-C, TC, TG, AIP, glucose, insulin, and HOMA-IR, together with increased HDL-C levels. Structural equation modeling revealed that these improvements were predominantly mediated through myokine-dependent pathways rather than direct group effects. In skeletal muscle tissue, both irisin and myonectin significantly influenced HDL-C, LDL-C, TG, TC, AIP, glucose, HOMA-IR, TMAO, IS, and S-equol; however, myonectin did not significantly affect TC and TMAO. Direct group effects on LDL-C, HOMA-IR, and IS were minimal, underscoring the central role of muscle-derived myokines in mediating these outcomes. In myocardial tissue, group influence on irisin was substantial, while the effect on myonectin was also significant. Irisin significantly modulated S-equol, HOMA-IR, and insulin, whereas myonectin affected S-equol, IS, HOMA-IR, and glucose. Moreover, direct group contributions to TC, TG, glucose, insulin, TMAO, IS, and S-equol were statistically significant. Overall, the effects observed in the myocardial model were primarily mediated via myonectin-dependent pathways, suggesting that group-level influences were largely transmitted through this mediator.

## Data Availability

The data generated and analyzed during the current study are available from the corresponding author upon reasonable request.

## Supplementary Materials

The following supporting information can be downloaded at: https://www.mdpi.com/article/10.3390/biomedicines13122853/s1, Table S1: Sequential multiple mediation analysis (PROCESS Model 6) evaluating the influence of group status on lipid, glycemic, and microbiota-related metabolites through irisin and myonectin derived from skeletal muscle and myocardial tissue.

## Abbreviations

The following abbreviations are used in this manuscript:

AIP	Atherogenic Index of Plasma
HDL-C	High-Density Lipoprotein Cholesterol
HICT	High-Intensity Continuous Training Group
IS	Indoxyl Sulfate
HOMA-IR	Homeostatic Model Assessment of Insulin Resistance
LDL-C	Low-Density Lipoprotein Cholesterol
MICT	Moderate-Intensity Continuous Training Group
TC	Total Cholesterol
TG	Triglycerides
TMAO	Trimethylamine N-oxide
PCA	Principal Component Analysis

## References

[1] Jukić I., Kolobarić N., Stupin A., Matić A., Kozina N., Mihaljević Z., Mihalj M., Šušnjara P., Stupin M., Ćurić Ž.B. (2021). Carnosine, Small but Mighty-Prospect of Use as Functional Ingredient for Functional Food Formulation. Antioxidants.

[2] Cesak O., Vostalova J., Vidlar A., Bastlova P., Student V. (2023). Carnosine and Beta-Alanine Supplementation in Human Medicine: Narrative Review and Critical Assessment. Nutrients.

[3] Everaert I., Stegen S., Vanheel B., Taes Y., Derave W. (2013). Effect of beta-alanine and carnosine supplementation on muscle contractility in mice. Med. Sci. Sports Exerc..

[4] Matsumoto T., Kojima M., Takayanagi K., Taguchi K., Kobayashi T. (2020). Role of S-Equol, Indoxyl Sulfate, and Trimethylamine N-Oxide on Vascular Function. Am. J. Hypertens..

[5] Mohr A.E., Jäger R., Carpenter K.C., Kerksick C.M., Purpura M., Townsend J.R., West N.P., Black K., Gleeson M., Pyne D.B. (2020). The athletic gut microbiota. J. Int. Soc. Sports Nutr..

[6] Monda V., Villano I., Messina A., Valenzano A., Esposito T., Moscatelli F., Viggiano A., Cibelli G., Chieffi S., Monda M. (2017). Exercise Modifies the Gut Microbiota with Positive Health Effects. Oxidative Med. Cell. Longev..

[7] Suryani D., Subhan Alfaqih M., Gunadi J.W., Sylviana N., Goenawan H., Megantara I., Lesmana R. (2022). Type, Intensity, and Duration of Exercise as Regulator of Gut Microbiome Profile. Curr. Sports Med. Rep..

[8] Torquati L., Gajanand T., Cox E.R., Willis C.R.G., Zaugg J., Keating S.E., Coombes J.S. (2023). Effects of exercise intensity on gut microbiome composition and function in people with type 2 diabetes. Eur. J. Sport Sci..

[9] Ticinesi A., Nouvenne A., Cerundolo N., Catania P., Prati B., Tana C., Meschi T. (2019). Gut Microbiota, Muscle Mass and Function in Aging: A Focus on Physical Frailty and Sarcopenia. Nutrients.

[10] Tian Z.-J., He Z.-X., Cai M.-X. (2013). Exercise intervention in skeletal muscle endocrine function. Sheng Li Ke Xue Jin Zhan Prog. Physiol..

[11] Kwon J.H., Moon K.M., Min K.W. (2020). Exercise-Induced Myokines can Explain the Importance of Physical Activity in the Elderly: An Overview. Healthcare.

[12] Mancinelli R., Checcaglini F., Coscia F., Gigliotti P., Fulle S., Fanò-Illic G. (2021). Biological Aspects of Selected Myokines in Skeletal Muscle: Focus on Aging. Int. J. Mol. Sci..

[13] Hoffmann C., Weigert C. (2017). Skeletal Muscle as an Endocrine Organ: The Role of Myokines in Exercise Adaptations. Cold Spring Harb. Perspect. Med..

[14] Mucher P., Batmyagmar D., Perkmann T., Repl M., Radakovics A., Ponocny-Seliger E., Lukas I., Fritzer-Szekeres M., Lehrner J., Knogler T. (2021). Basal myokine levels are associated with quality of life and depressed mood in older adults. Psychophysiology.

[15] Alves H.R., Lomba G.S.B., Gonçalves-de-Albuquerque C.F., Burth P. (2022). Irisin, Exercise, and COVID-19. Front. Endocrinol..

[16] Demir İ., Guler A. (2020). Association of decreased myonectin levels with metabolic and hormonal disturbance in polycystic ovary syndrome. Gynecol. Endocrinol..

[17] Foright R.M., Johnson G.C., Kahn D., Charleston C.A., Presby D.M., Bouchet C.A., Wellberg E.A., Sherk V.D., Jackman M.R., Greenwood B.N. (2020). Compensatory eating behaviors in male and female rats in response to exercise training. Am. J. Physiol. Regul. Integr. Comp. Physiol..

[18] Barsky S.T., Monks D.A. (2020). Myocytic androgen receptor overexpression does not affect sex differences in adaptation to chronic endurance exercise. Biol. Sex Differ..

[19] Schaalan M.F., Ramadan B.K., Abd Elwahab A.H. (2018). Synergistic effect of carnosine on browning of adipose tissue in exercised obese rats; a focus on circulating irisin levels. J. Cell. Physiol..

[20] Kim S.H., Cho Y.K., Kim Y.J., Jung C.H., Lee W.J., Park J.Y., Huh J.H., Kang J.G., Lee S.J., Ihm S.H. (2022). Association of the atherogenic index of plasma with cardiovascular risk beyond the traditional risk factors: A nationwide population-based cohort study. Cardiovasc. Diabetol..

[21] Jia Y., Song T., Li Z., Zhou L., Chen S. (2022). The Relationship Between Triglyceride Glucose Index and Vitamin D in Type 2 Diabetes Mellitus. Diabetes Metab. Syndr. Obes..

[22] Park S.Y., Gautier J.F., Chon S. (2021). Assessment of Insulin Secretion and Insulin Resistance in Human. Diabetes Metab. J..

[23] Zeng N., Liao T., Chen X.Y., Yan Z.P., Li J.T., Ni G.X. (2021). Treadmill running induces remodeling of the infrapatellar fat pad in an intensity-dependent manner. J. Orthop. Surg. Res..

[24] Soya H., Mukai A., Deocaris C.C., Ohiwa N., Chang H., Nishijima T., Fujikawa T., Togashi K., Saito T. (2007). Threshold-like pattern of neuronal activation in the hypothalamus during treadmill running: Establishment of a minimum running stress (MRS) rat model. Neurosci. Res..

[25] Yasul Y., Akçınar F., Çınar V., Akbulut T., Aydemir İ., Yalçın M.H., Avcu E.Ç., Aydın S., Aydın S. (2025). Moderate/high-intensity exercise and coenzyme q10 supplementation may reduce tumstatin and improve the lipid dynamics and body mass in rats. Appl. Sci..

[26] Bolat H., Akbulut T., Çınar V., Avcu E.Ç., Yasul Y., Bozbay K., Aydın S. (2025). Modulation of angiogenesis-related biomarkers in brain and myocardial tissues by exercise and carnosine: An ELISA-based analysis of VEGF-A, HIF-1α, Ang-1, MMP-9, and anti-angiogenic factors. Mol. Nutr. Food Res..

[27] Hegazy M.A., Abdelmonsif D.A., Zeitoun T.M., El-Sayed N.S., Samy D.M. (2022). Swimming exercise versus L-carnosine supplementation for Alzheimer’s dementia in rats: Implication of circulating and hippocampal FNDC5/irisin. J. Physiol. Biochem..

[28] Aydın S., Ogeturk M., Kuloglu T., Kavakli A., Aydin S. (2015). Effect of carnosine supplementation on apoptosis and irisin, total oxidant and antioxidant levels in the serum, liver and lung tissues in rats exposed to formaldehyde inhalation. Peptides.

[29] Rašković A., Martić N., Zaklan D., Duborija-Kovačević N., Vujčić M., Andrejić-Višnjić B., Čapo I., Mijović R., Krga M., Pavlović N. (2023). Antihyperlipidemic potential of dietary supplementation with carnosine in high-fat diet-fed rats. Eur. Rev. Med. Pharmacol. Sci..

[30] Hsu S.M., Raine L., Fanger H. (1981). Use of avidin-biotin-peroxidase complex (ABC) in immunoperoxidase techniques: A comparison between ABC and unlabeled antibody (PAP) procedures. J. Histochem. Cytochem. Off. J. Histochem. Soc..

[31] Avcu E.C., Çınar V., Yasul Y., Akbulut T., Pancar Z., Aydemir I.S., Aydin S., Yalcin M.H., Aydin S. (2024). Effects of an energy drink on myonectin in the liver, kidney and skeletal muscle of exercised rats. Biotech. Histochem. Off. Publ. Biol. Stain. Comm..

[32] Yılmaz B., Şenel Ö., Çalıkuşu A., Abbasoğlu E.G., Yasul Y., Anadol E., Sarısoy F., Atalar K., Bahçelioğlu M., Yılmaz C. (2025). CoQ10-Supported HIIT Modulates Skeletal Muscle and Hippocampal Biomarkers in Rats: A Randomized, Repeated-Measures, Post-Test Controlled Design. Antioxidants.

[33] Yasul Y., Yılmaz B., Şenel Ö., Kurt D., Akbulut T., Çalıkuşu A., Anadol E., Yılmaz C. (2025). Evaluating the impact of coenzyme Q10 and high-intensity interval training on lactate threshold and Plasma blood gases in rats: A randomized controlled trial. Eur. J. Appl. Physiol..

[34] Yiğiter N., Akçınar F., Yasul Y., Çınar V., Akbulut T., Migliaccio G.M. (2025). Core Exercise as Non-Pharmacological Strategy for Improving Metabolic Health in Prediabetic Women. Medicina.

[35] Hayes A.F. (2018). Introduction to Mediation, Moderation, and Conditional Process Analysis: A Regression-Based Approach.

[36] Cohen J. (2013). Statistical Power Analysis for the Behavioral Sciences.

[37] Hoene M., Li J., Li Y., Runge H., Zhao X., Häring H.U., Lehmann R., Xu G., Weigert C. (2016). Muscle and liver-specific alterations in lipid and acylcarnitine metabolism after a single bout of exercise in mice. Sci. Rep..

[38] Trevellin E., Scorzeto M., Olivieri M., Granzotto M., Valerio A., Tedesco L., Fabris R., Serra R., Quarta M., Reggiani C. (2014). Exercise training induces mitochondrial biogenesis and glucose uptake in subcutaneous adipose tissue through eNOS-dependent mechanisms. Diabetes.

[39] Rahim H.A., Damirchi A., Babaei P. (2024). Comparison of HIIT and MICT and further detraining on metabolic syndrome and asprosin signaling pathway in metabolic syndrome model of rats. Sci. Rep..

[40] Mattioni Maturana F., Martus P., Zipfel S., Nieß A.M. (2021). Effectiveness of HIIE versus MICT in Improving Cardiometabolic Risk Factors in Health and Disease: A Meta-analysis. Med. Sci. Sports Exerc..

[41] Ravi Kiran T., Subramanyam M.V., Prathima S., Asha Devi S. (2006). Blood lipid profile and myocardial superoxide dismutase in swim-trained young and middle-aged rats: Comparison between left and right ventricular adaptations to oxidative stress. J. Comp. Physiol. B Biochem. Syst. Environ. Physiol..

[42] Kim M.Y., Kim E.J., Kim Y.N., Choi C., Lee B.H. (2011). Effects of α-lipoic acid and L-carnosine supplementation on antioxidant activities and lipid profiles in rats. Nutr. Res. Pract..

[43] Nagai K., Niijima A., Yamano T., Otani H., Okumra N., Tsuruoka N., Nakai M., Kiso Y. (2003). Possible role of L-carnosine in the regulation of blood glucose through controlling autonomic nerves. Exp. Biol. Med..

[44] Nagai K., Tanida M., Niijima A., Tsuruoka N., Kiso Y., Horii Y., Shen J., Okumura N. (2012). Role of L-carnosine in the control of blood glucose, blood pressure, thermogenesis, and lipolysis by autonomic nerves in rats: Involvement of the circadian clock and histamine. Amino Acids.

[45] Aydın A.F., Bingül İ., Küçükgergin C., Doğan-Ekici I., Doğru Abbasoğlu S., Uysal M. (2017). Carnosine decreased oxidation and glycation products in serum and liver of high-fat diet and low-dose streptozotocin-induced diabetic rats. Int. J. Exp. Pathol..

[46] Cripps M.J., Hanna K., Lavilla C., Sayers S.R., Caton P.W., Sims C., De Girolamo L., Sale C., Turner M.D. (2017). Carnosine scavenging of glucolipotoxic free radicals enhances insulin secretion and glucose uptake. Sci. Rep..

[47] Yang L., Lin W., Yan X., Zhang Z. (2024). Comparative effects of lifelong moderate-intensity continuous training and high-intensity interval training on blood lipid levels and mental well-being in naturally ageing mice. Exp. Gerontol..

[48] Kazeminasab F., Marandi M., Ghaedi K., Esfarjani F., Moshtaghian J. (2017). Effects of A 4-Week Aerobic Exercise on Lipid Profile and Expression of LXRα in Rat Liver. Cell J..

[49] Coll-Risco I., Aparicio V.A., Nebot E., Camiletti-Moirón D., Martínez R., Kapravelou G., López-Jurado M., Porres J.M., Aranda P. (2016). Effects of interval aerobic training combined with strength exercise on body composition, glycaemic and lipid profile and aerobic capacity of obese rats. J. Sports Sci..

[50] Chang G.R., Hou P.H., Chen W.K., Lin C.T., Tsai H.P., Mao F.C. (2020). Exercise Affects Blood Glucose Levels and Tissue Chromium Distribution in High-Fat Diet-Fed C57BL6 Mice. Molecules.

[51] Barretti D.L.M., Magalhaes F.D.C., Fernandes T., do Carmo E.C., Rosa K.T., Irigoyen M.C., Negrao C.E., Oliveira E.M. (2012). Effects of aerobic exercise training on cardiac renin-angiotensin system in an obese Zucker rat strain. PLoS ONE.

[52] Akbulut T., Cinar V., Aydin S., Yardim M. (2022). The role of different exercises in irisin, heat shock protein 70 and some biochemical parameters. J. Med. Biochem..

[53] Hoseini R., Rahim H.A., Ahmed J.K. (2022). Decreased inflammatory gene expression accompanies the improvement of liver enzyme and lipid profile following aerobic training and vitamin D supplementation in T2DM patients. BMC Endocr. Disord..

[54] Gallo-Villegas J., Aristizabal J.C., Estrada M., Valbuena L.H., Narvaez-Sanchez R., Osorio J., Aguirre-Acevedo D.C., Calderón J.C. (2018). Efficacy of high-intensity, low-volume interval training compared to continuous aerobic training on insulin resistance, skeletal muscle structure and function in adults with metabolic syndrome: Study protocol for a randomized controlled clinical trial (Intraining-MET). Trials.

[55] Cui J., Bai Y., Li M., Xu X., Dai Y., Zhang J. (2014). Effects of different intensity exercise on blood glucose, adolescent obesity rats insulin sensitivity and RBP4. Wei Sheng Yan Jiu J. Hyg. Res..

[56] Costa J.S.R., Fonseca G., Ottone N., Silva P.A., Antonaccio R.F., Silva G., Rocha M., Coimbra C.C., Esteves E.A., Mang Z.A. (2021). Strength training improves insulin resistance and differently affects mitochondria in skeletal muscle and visceral adipose tissue in high-fat fed mice. Life Sci..

[57] Forsberg E.A., Botusan I.R., Wang J., Peters V., Ansurudeen I., Brismar K., Catrina S.B. (2015). Carnosine decreases IGFBP1 production in db/db mice through suppression of HIF-1. J. Endocrinol..

[58] Al-Sawalha N.A., Alshogran O.Y., Awawdeh M.S., Almomani B.A. (2019). The effects of l-Carnosine on development of metabolic syndrome in rats. Life Sci..

[59] Albrecht T., Schilperoort M., Zhang S., Braun J.D., Qiu J., Rodriguez A., Pastene D.O., Krämer B.K., Köppel H., Baelde H. (2017). Carnosine Attenuates the Development of both Type 2 Diabetes and Diabetic Nephropathy in BTBR ob/ob Mice. Sci. Rep..

[60] Magkos F., Fraterrigo G., Yoshino J., Luecking C., Kirbach K., Kelly S.C., de Las Fuentes L., He S., Okunade A.L., Patterson B.W. (2016). Effects of moderate and subsequent progressive weight loss on metabolic function and adipose tissue biology in humans with obesity. Cell Metab..

[61] Pedersen B.K., Febbraio M.A. (2012). Muscles, exercise and obesity: Skeletal muscle as a secretory organ. Nat. Rev. Endocrinol..

[62] Wu Y., Yang Y., Zhong Y., Wu Y., Zhang Z., Yan Z., Wang W. (2024). Unveiling the dynamic processes of dietary advanced glycation end-products (dAGEs) in absorption, accumulation, and gut microbiota metabolism. Food Funct..

[63] Caruso G., Benatti C., Musso N., Fresta C.G., Fidilio A., Spampinato G., Brunello N., Bucolo C., Drago F., Lunte S.M. (2021). Carnosine protects macrophages against the toxicity of Aβ1–42 oligomers by decreasing oxidative stress. Biomedicines.

[64] Aldini G., de Courten B., Regazzoni L., Gilardoni E., Ferrario G., Baron G., Carini M. (2021). Understanding the antioxidant and carbonyl sequestering activity of carnosine: Direct and indirect mechanisms. Free Radic. Res..

[65] Pinto-Junior D.C., Silva K.S., Michalani M.L., Marinho R., Fortes M.A.S., Curi R., Ferreira F., Velloso L.A., Homem de Bittencourt P.I., Pauli J.R. (2018). Advanced glycation end products-induced insulin resistance involves repression of skeletal muscle GLUT4 expression. Sci. Rep..

[66] Valentino T.R., Vechetti I.J., Mobley C.B., Dungan C.M., Golden L., Goh J., McCarthy J.J. (2021). Dysbiosis of the gut microbiome impairs mouse skeletal muscle adaptation to exercise. J. Physiol..

[67] Scheiman J., Luber J.M., Chavkin T.A., MacDonald T., Tung A., Pham L.D., Wibowo M.C., Wurth R.C., Punthambaker S., Tierney B.T. (2019). Meta-omics analysis of elite athletes identifies a performance-enhancing microbe that functions via lactate metabolism. Nat. Med..

[68] Taesuwan S., Cho C.E., Malysheva O.V., Bender E., King J.H., Yan J., Thalacker-Mercer A.E., Caudill M.A. (2017). The metabolic fate of isotopically labeled trimethylamine-N-oxide (TMAO) in humans. J. Nutr. Biochem..

[69] Zou H., Zhou Y., Gong L., Huang C., Liu X., Lu R., Yu J., Kong Z., Zhang Y., Lin D. (2024). Trimethylamine N-Oxide Improves Exercise Performance by Reducing Oxidative Stress through Activation of the Nrf2 Signaling Pathway. Molecules.

[70] Pechlivanis A., Papaioannou K.G., Tsalis G., Saraslanidis P., Mougios V., Theodoridis G.A. (2015). Monitoring the Response of the Human Urinary Metabolome to Brief Maximal Exercise by a Combination of RP-UPLC-MS and (1)H NMR Spectroscopy. J. Proteome Res..

[71] Enea C., Seguin F., Petitpas-Mulliez J., Boildieu N., Boisseau N., Delpech N., Diaz V., Eugène M., Dugué B. (2010). (1)H NMR-based metabolomics approach for exploring urinary metabolome modifications after acute and chronic physical exercise. Anal. Bioanal. Chem..

[72] Almer G., Semeraro M.D., Meinitzer A., Enko D., Rodriguez Blanco G., Gallé B., Horvath A., Moissl-Eichinger C., Till H., Gruber H.-J. (2023). Impact of long-term high dietary fat intake and regular exercise on serum TMAO and microbiome composition in female rats. Nutr. Healthy Aging.

[73] Saadati S., de Courten M., Deceneux C., Plebanski M., Scott D., Mesinovic J., Jansons P., Aldini G., Cameron J., Feehan J. (2024). Carnosine Supplementation Has No Effect on Inflammatory Markers in Adults with Prediabetes and Type 2 Diabetes: A Randomised Controlled Trial. Nutrients.

[74] Sekikawa A., Ihara M., Lopez O., Kakuta C., Lopresti B., Higashiyama A., Aizenstein H., Chang Y.F., Mathis C., Miyamoto Y. (2019). Effect of S-equol and Soy Isoflavones on Heart and Brain. Curr. Cardiol. Rev..

[75] Hayashi K., Yamaguchi H., Amaoka H., Takahara T., Kunisa S., Tamai N., Maejima N., Watanabe N., Kobayashi Y., Tanaka H. (2021). Equol-producing status affects exercise training-induced improvement in arterial compliance in postmenopausal women. J. Appl. Physiol..

[76] Cheng P.F., Chen J.J., Zhou X.Y., Ren Y.F., Huang W., Zhou J.J., Xie P. (2015). Do soy isoflavones improve cognitive function in postmenopausal women? A meta-analysis. Menopause.

[77] Leong S.C., Sirich T.L. (2016). Indoxyl Sulfate-Review of Toxicity and Therapeutic Strategies. Toxins.

[78] Graboski A.L., Kowalewski M.E., Simpson J.B., Cao X., Ha M., Zhang J., Walton W.G., Flaherty D.P., Redinbo M.R. (2023). Mechanism-based inhibition of gut microbial tryptophanases reduces serum indoxyl sulfate. Cell Chem. Biol..

[79] Nishikawa M., Ishimori N., Takada S., Saito A., Kadoguchi T., Furihata T., Fukushima A., Matsushima S., Yokota T., Kinugawa S. (2015). AST-120 ameliorates lowered exercise capacity and mitochondrial biogenesis in the skeletal muscle from mice with chronic kidney disease via reducing oxidative stress. Nephrol. Dial. Transplant. Off. Publ. Eur. Dial. Transpl. Assoc.-Eur. Ren. Assoc..

[80] Enoki Y., Watanabe H., Arake R., Fujimura R., Ishiodori K., Imafuku T., Nishida K., Sugimoto R., Nagao S., Miyamura S. (2017). Potential therapeutic interventions for chronic kidney disease-associated sarcopenia via indoxyl sulfate-induced mitochondrial dysfunction. J. Cachexia Sarcopenia Muscle.

[81] Cheng T.H., Ma M.C., Liao M.T., Zheng C.M., Lu K.C., Liao C.H., Hou Y.C., Liu W.C., Lu C.L. (2020). Indoxyl Sulfate, a Tubular Toxin, Contributes to the Development of Chronic Kidney Disease. Toxins.

