# Supplementary material for: Exercise and Carnosine Modulate Microbiota-Derived Metabolites, Myokines, and Cardiometabolic Profiles in Rats: A Randomized Controlled Trial
Source: Biomedicines. 2025 Nov 22;13(12):2853. doi: 10.3390/biomedicines13122853 (PMC12730646; doi:10.3390/biomedicines13122853)
Supplement: Supplementary file 1 [file biomedicines-13-02853-s001.zip › biomedicines-3909941-supplementary.pdf]

SUPPLEMENTARY MATERIALS

Full Title: Exercise and Carnosine Modulate Microbiota-Derived Metabolites, Myokines, and Cardiometabolic Profiles in Rats: A Randomized Controlled Trial

Authors: Kenan Bozbay [1], Vedat Çınar [1], Taner Akbulut [1*], Yavuz Yasul [2], Mehmet Hanifi Yalçın [3], Meva Ceren Orgun [1], Süleyman Aydın [4], Do-Youn Lee [5,*]

Affiliations:

[1] School of Physical Education and Sports, Dicle University, Diyarbakır, Turkiye
[2] Ondokuz Mayıs University, Bafra Vocational School, Samsun, Turkiye
[3] Department of Histology and Embryology, Faculty of Veterinary Medicine, Fırat University, Elazig, Turkiye
[4] Faculty of Medicine, Department of Medical Biochemistry, Fırat University, Elazig, Turkiye
[5] College of General Education, Kookmin University, Seoul, Korea

* Correspondence: takbulut@firat.edu.tr (T.A.), triptoyoun@kookmin.ac.kr (DY.L)

Supplemental Table S1. Sequential multiple mediation analysis (PROCESS Model 6) evaluating the influence of group status on lipid, glycemic, and microbiota-related metabolites through irisin and myonectin derived from skeletal muscle and myocardial tissue

Dependent variables	a_1 (beta)	b_1 (beta)	a_2 (beta)	d_{21} (beta)	b_2 (beta)	c (beta) Total effect	c′cs (beta) Direct effect	Completely standardised indirect effet 95% CI			Total indirect effect 95% CI
(Y)	($X \to M_1$)	($M_1 \to Y$)	($X \to M_2$)	($M_1 \to M_2$)	($M_2 \to Y$)	($X \to Y$)	($X \to Y$)	a_1b_1 ($X \to M_1 \to Y$)	a_2b_2 ($X \to M_2 \to Y$)	$a_1d_{21}b_2$ ($X \to M_1 \to M_2 \to Y$)	
**Skeletal muscle tissue results**											
**HDL-C**	.887***	.465**	.477**	.435**	.558***	.770***	–.124	.413 [.131, .687]	.266 [.069, .566]	.215 [.053, .433]	.894 [.597, 1.252]
**LDL-C**	.887***	-.598***	.477**	.435**	-.592***	-.780***	.262*	-.531 [-.752, -.302]	-.282 [-.509, -.102]	-.228 [-.431, -.054]	-1.042 [-1.256, -.843]
**TC**	.887***	-.516*	.477**	.435**	-.158	-.767***	-.172	-.165 [-.789, -.143]	-.075 [-.310, -.096]	-.061 [-.206, .931]	-.594 [-.929, -.287]
**TG**	.887***	-.495*	.477**	.435**	-.409*	-.769***	.022	-.439 [-.747, -.137]	-.195 [-.474, -.011]	-.157 [-.347, .005]	-.792 [-1.218, -.403]
**AIP**	.887***	-.376*	.477**	.435**	-.513**	-.801***	-.024	-.333 [-.594, -.077]	-.245 [-.543, -.062]	-.198 [-.399, .032]	-.776 [-1.142, -.424]
**Glucose**	.887***	-.366*	.477**	.435**	-.464**	-.809***	-.083	-.325 [-.591, -.049]	-.221 [-.490, -.042]	-.179 [-.346, .031]	-.725 [-1.022, -.443]
**Insulin**	.887***	-.293	.477**	.435**	-.516**	-.780***	-.073	-.260 [-.626, -.106]	-.246 [-.498, -.063]	-.199 [-.434, .036]	-.706 [-1.063, -.365]
**HOMA-IR**	.887***	-.296*	.477**	.435**	-.506**	-.843***	-.143)	-.263 [-.521, .003]	-.242 [-.463, -.077]	-.195 [-.389, -.040]	-.700 [-.954, -.438]
**TMAO**	.887***	-.631**	.477**	.435**	-.188	-.767***	-.045	-.560 [-.902, -.146]	-.090 [-.283, .109]	-.073 [-.318, .049]	-.722 [-1.011, -.392]
**IS**	.887***	.780**	.477**	.435**	.631*	.069	-1.168***	.692 [.203, 1.125]	.301 [.056, .677]	.243 [.026, .557]	1.236 [.812, 1.726]
**S-equol**	.887***	.548**	.477**	.435**	.427**	.801***	–.054	.487 [.156, .752]	.204 [.040, .419]	.165 [.019, .380]	.855 [.567, 1.111]
**Myocardial tissue results**											
**HDL-C**	.812***	.473***	.266*	.609**	.412***	.770***	.270	.384 [.126, 0.639]	.109 [.005, .268]	.204 [.054, .386]	.698 [.531,.870]
**LDL-C**	.812***	-.538***	.266*	.609***	-.279*	-.780***	-.131	-.437 [-.688, -.133]	-.074 [-.216, .001]	-.138 [-.306, -.012]	-.650 [-.820, -.453]
**TC**	.812***	-.303	.266*	.609***	-.321*	-.768***	-.278	-.246 [-.542, .036]	-.085 [-.235, .001]	-.159 [-.322, -.020]	-.490 [-.722, -.286]
**TG**	.812***	-.593***	.266	.609***	-.046	-.769***	-.253	-.482 [-.711, -.224]	-.012 [-.102, .083]	-.023 [-.177, .111]	-.517 [-.747, -.229]
**AIP**	.812***	-.523***	.266	.609***	-.261*	-.801***	-.178	-.425 [-.648, -.171]	-.069 [-.173, .002]	-.129 [-.288, -.016]	-.623 [-.802, -.432]
**Glucose**	.812***	-.250	.266	.609***	-.275	-.809***	-.397**	-.203 [-.442, .058]	-.073 [-.210, .003]	-.136 [-.284, -.009]	-.412 [-.645, -.203]
**Insulin**	.812***	-.347*	.266	.609***	-.184	-.780***	-.358*	-.282 [-.543, -.001]	-.049 [-.173, .030]	-.091 [-.237, .062]	-.422 [-.660, -.167]
**HOMA-IR**	.812***	-.314*	.266	.609***	-.225	-.843***	-.417**	-.255 [-.477, -.023]	-.060 [-.176, .006]	-.111 [-.235, .008]	-.426 [-.621, -.235]
**TMAO**	.812***	-.278	.266	.609***	-.250	-.767***	-.352*	-.225 [-.514, .082]	-.066 [-.217, .019]	-.123 [-.304, .036]	-.415 [-.641, -.188]
**IS**	.812***	.354	.266	.609***	.845	.069	-.861***	.288 [-.176, .703]	.225 [.019, .541]	418 [.154, .764]	.930 [.606, 1.283]
**S-equol**	.812***	.374*	.266	.609***	.280*	.801***	.284*	.304 [.021, .557]	.075 [-.003, .203]	.139 [.006, .307]	.517 [.304, .712]

Standardized total, direct, and indirect effects of group status (X) on lipid markers (HDL-C, LDL-C, TC, TG, AIP; Y), glycemic indicators (glucose, insulin, HOMA-IR; Y), and microbiota-related metabolites (TMAO, IS, S-equol; Y) through irisin (M1) and myonectin (M2) derived from skeletal muscle and myocardial tissue are presented. Indirect effects were estimated using 5000 bootstrap resamples with 95 percent confidence intervals. HDL-C: high-density lipoprotein cholesterol, LDL-C: low-density lipoprotein cholesterol, TC: total cholesterol, TG: triglycerides, AIP: atherogenic index of plasma, HOMA-IR: homeostatic model assessment of insulin resistance, TMAO: trimethylamine-N-oxide, IS: indoxyl sulfate, X: independent variable (groups); Y: dependent variables; M1/M2: sequential mediators; CI: confidence interval, *: $p<0.05$, **: $p<0.01$, ***: $p<0.001$.
